# The Impact of the Land Cover Dynamics on Surface Urban Heat Island Variations in Semi-Arid Cities: A Case Study in Ahmedabad City, India, Using Multi-Sensor/Source Data

**DOI:** 10.3390/s19173701

**Published:** 2019-08-26

**Authors:** Pir Mohammad, Ajanta Goswami, Stefania Bonafoni

**Affiliations:** 1 Department of Earth Sciences, IIT Roorkee, Uttarakhand 247667, India; 2 Department of Engineering, University of Perugia, via Duranti 93, 06125 Perugia, Italy

**Keywords:** surface urban heat island, semi-arid city, MODIS, land surface temperature, rural area, land cover, white sky albedo, evapotranspiration, groundwater table level, SUHI footprint

## Abstract

This study examines the behavior of land surface temperature (LST) and surface urban heat island (SUHI) from MODIS data over Ahmedabad city, Gujarat state (India), from 2003 to 2018. Summer and winter LST patterns were analyzed, both daytime and nighttime. Ahmedabad, one of the fastest growing metropolitan cities in India, is characterized by a semi-arid climate. The investigation focuses on the SUHI variations due to warming or cooling trends of both urban and rural areas, providing quantitative interpretations by means of multi-sensor/source data. Land cover maps, normalized differential vegetation index, surface albedo, evapotranspiration, urban population, and groundwater level were analyzed across the years to assess their impact on SUHI variations. Moreover, a field campaign was carried out in summer 2018 to measure LST in several rural and urban sites. During summer daytime, the rural zone exhibits a higher average LST than the urban area, resulting in a mean negative SUHI, typical of arid cities, while a slight positive SUHI (mean intensity of 0.4 °C) during winter daytime is present. An evident positive SUHI is found only during summer (1.8 °C) and winter nighttime (3.2 °C). The negative SUHI intensity is due to the low vegetation presence in the rural area, dominated by croplands turning into bare land surfaces during the pre-monsoon summer season. Higher LST values in the rural area than in the urban area are also confirmed by the field campaign, with an average difference of about 5 °C. Therefore, the impact of the rural LST in biasing the SUHI is evident, and a careful biophysical interpretation is needed. For instance, within the urban area, the yearly intensity of the summer daytime SUHI is not correlated with the evapotranspiration, while the correspondent summer daytime LST exhibits a significant negative correlation (−0.73) with evapotranspiration. Furthermore, despite the city growth across the years, the urban area does not generally reveal a temporal increase of the magnitude of the heat island but an enlargement of its spatial footprint.

## 1. Introduction

Environmental and local climate effects of land cover and land use changes over the years are clearly visible in the urbanization process. For instance, the conversion of natural areas to impervious surfaces results in a decrease of the evapotranspiration and in an enhancement of absorption and trapping of solar radiation in the urban areas, causing the well-known urban heat island (UHI) phenomenon [1,2]. The UHI intensity, quantified as the difference in air or surface temperature between the urban area and rural surroundings, is gaining importance with time, since extreme heat wave conditions occurring in the urban area can lead to dangerous, even deadly, health consequences, including heat stress and heatstroke [3,4,5,6]. 

The UHI pattern can be influenced by external factors (urban area location and climate conditions) and intrinsic factors (land use and land cover, city size and growth, human activities) [7]. Changes of the intrinsic factors mainly affect the land surface temperature (LST) dynamics over the urban area, and potentially explain the surface urban heat island (SUHI) variations. For instance, heat released from energy consumption, changes in materials, and the albedo of pavements and building rooftops [8,9] can modify the SUHI intensity (SUHII) during the years. The increase of green spaces in the urban texture is a well-known strategy to mitigate the SUHI effects [10,11]. 

Satellite-based thermal data is frequently used for the assessment of the SUHI instead of using in situ measurements [12], which supply sparsely distributed data. Satellite sensors can provide reliable and repeatable Earth surface observations, allowing a study of the urban environment at various spatial and temporal scales. A thorough and comprehensive review of SUHI using spaceborne sensors is provided in [12], including a literature overview of the SUHII variations at a local scale driven by land cover, land use and their changes, urban site characteristics, landscape composition, and configuration. The use of remote sensing techniques and surface ancillary data at different spatial and temporal scales is also useful in providing indications to regulate urban planning policy [13,14].

Although SUHI dynamics are generally associated with urban sprawl, land cover and land use changes, increasing industrial and human activities, and urban materials, in arid and semi-arid climates, the seasonality and the diurnal cycle can generate different effects on the SUHI [15]. Indeed, variations of SUHII across seasons or years are a consequence of warming/cooling trends of both urban and rural areas. For instance, an SUHI intensification can be caused by a cooling trend in the rural areas albeit a weak heating is observed in the urban zones, as well as an SUHI mitigation due to a warming trend of the rural areas instead of urban heat alleviation strategies. 

Arid and semi-arid cities exhibit an inversion of the typical SUHI phenomenon in daytime, with the city core appearing cooler compared to the suburbs and rural surroundings [16]. Cooling effects have been found in arid cities during the summer months in different parts of the world [17,18,19,20]. 

Lazzarini et al. [16] used LST from different satellite sensors (MODIS, ASTER, and Landsat 7) to analyze the SUHI in Abu Dhabi at different spatial scales, exploring the LST relationships with the normalized difference vegetation index (NDVI) and the impervious surface areas (ISA). They found that the urban area appeared cooler compared to the suburbs, with the negative SUHI more accentuated in summer than winter. Haashemi et al. [15] investigated the seasonal variability of SUHI in Tehran, Iran using MODIS and Landsat 8 thermal data. They related SUHI intensity with different potential drivers, i.e., land use and land cover (LULC), elevation, ISA, fractional vegetation cover (FVC), and albedo. Their result showed the existence of a negative heat island at daytime, but the relationship with the selected drivers possessed large seasonal variations. Frey et al. [21] found a daytime surface cool island in two coastal cities, Dubai and Abu Dhabi, using ASTER data. They related the surface temperature variation with albedo, emissivity, and net radiation in different urban and rural land cover classes. In Rasul et al. [17], a review of different works dealing with surface urban cool island (SUCI) in different semi-arid cities is provided. The authors highlighted how few works have investigated the spatiotemporal variation of surface SUHI/SUCI and the effect of LULC changes on LST in arid and semi-arid climates, suggesting more in-depth studies in these areas. 

In India, during summer pre-monsoon months, different cities behave like an arid or semi-arid region and hence are characterized by negative urban–rural LST differences [22]. Our study focuses on the analysis of the SUHI of Ahmedabad city, Gujarat state (India), one of the fastest growing metropolitan cities in India characterized by a semi-arid climate. Ahmedabad suffered a relevant heat wave during summer 2010, with record temperatures and health consequences [23]. In this scenario, a deep investigation of the diurnal, seasonal, and annual behavior of the SUHI was carried out. Even though a positive SUHI over Ahmedabad is discussed in different papers [24,25,26], the analysis of its negative SUHI on summer daytime is still missing.

The proposed study points out the relevance of quantifying LST changes and trends in both urban and rural areas to provide a better insight of the SUHII spatial and temporal variations. In this way, a clearer interpretation is provided when summer/winter or daytime/nighttime SUHI analyses reveal opposite behaviors, as in arid and semi-arid cities. Specifically, the rate at which the Ahmedabad area is changing in term of LST and SUHII over a period of 16 years (2003–2018) was evaluated using MODIS measurements data. MODIS LST of a 1-km spatial resolution provides a way of studying the urban heat phenomenon at diurnal, monthly, and seasonal scales [27,28,29,30,31,32]. Land cover maps were analyzed across the years, as well as the changes of further parameters: SUHI footprint, vegetation index, surface albedo, evapotranspiration, urban population, and groundwater level. Moreover, a field campaign was carried out in summer 2018 to measure LST in several rural and urban sites (158 points) to assess with in situ sensors the different thermal behaviors of the urban/non-urban regions. 

The main novelty of this study is the SUHI investigation in a semi-arid city, with data from different sources and sensors (both space-based and ground-based), not yet explored together in the literature. Specifically, spaceborne products from a MODIS sensor (LST, NDVI, albedo) were used: LULC from the Climate Change Initiative (CCI) of the European Space Agency (ESA), population data from the World Urbanization Prospects (WUP), evapotranspiration data from the operational simplified surface energy balance (SSEB) model, groundwater table level (WT) data from the Central Water Commission (CWC), and in situ LST measurements from an infrared thermometer. It is important to note that, for the first time, groundwater consumption time series were used in the SUHI and LST analysis. Overall, this study goes in the direction suggested in [17], i.e., an in-depth spatiotemporal analysis of the urban heat island in semi-arid climates.

## 2. Materials and Methods 

### 2.1. Study Area

**Figure 1:**
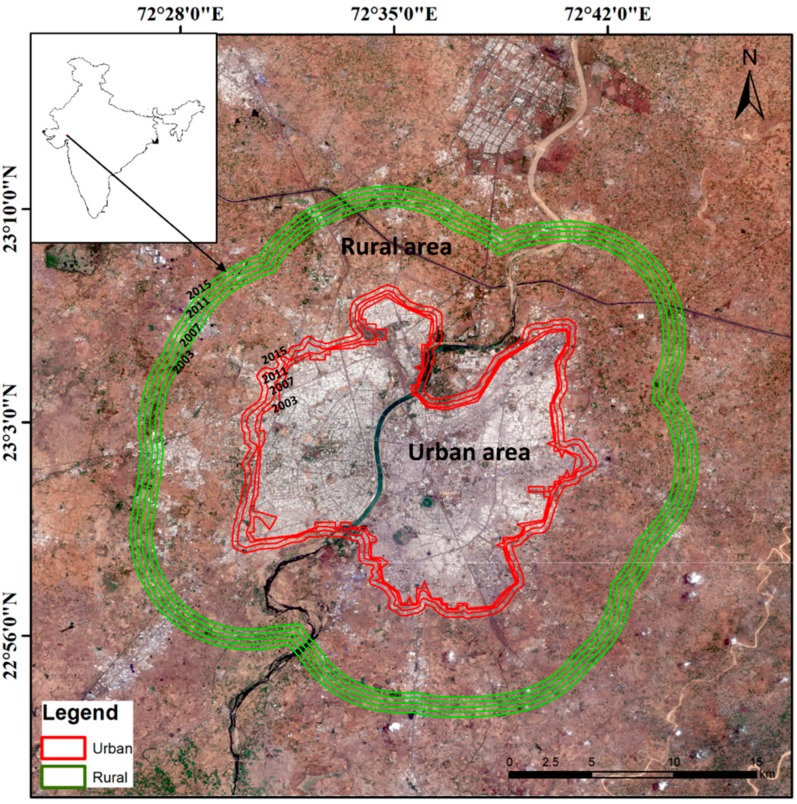
Map of Ahmedabad city showing urban (red) and rural (green) boundaries selected for the years 2003, 2007, 2011, and 2015 (true color composite map from Sentinel 2A on 2 March 2017).

Ahmedabad is a metropolis of the Gujarat state (Northwestern coast of India), crossed by the Sabarmati River (Figure 1). It is one of the seven largest cities of India, with a population of 7.2 million [33]. It is characterized by a hot, semi-arid climate, according to the Köppen climate classification [34]. 

The hottest months are from March to June, with an average maximum and minimum air temperature of 42 °C and 28 °C, respectively, while during the winter season, these temperatures are 30 °C and 15 °C. The mean annual rainfall over the city area is 782 mm. The land use percentages for residential and commercial categories are currently 44% and 3.4%, respectively, which were 35% and 2.5% in 1997. 

To compute the SUHII, the urban boundary has been yearly extracted from the 300 m annual global land cover time series of the Climate Change Initiative (CCI) of the European Space Agency (ESA). Urban boundaries for the year 2003, 2007, 2011, and 2015 are illustrated in Figure 1 (red curves), while the green curves roughly sketch the non-urban buffers of about 5 km surrounding the urban area, considered as rural area for the SUHI computation (see Section 2.3.2).

### 2.2. Data Source

Effective analysis of urban heating requires a large geospatial database over several years. Firstly, we considered the LST products from MODIS, on board Terra and Aqua satellites. The MODIS sensor measures radiation in 36 spectral bands (from 0.4 to 14.4 µm), providing different spatial resolution data (two bands at 250 m, five at 500 m, and 29 bands at 1 km). The daytime overpass over the study area for Terra and Aqua is around 10:30 and 13:30 local solar time, respectively, while during nighttime it is around 22:30 and 01:30 local solar time, respectively.

Global LST 8-day composite at a 1-km resolution (MYD11A2 and MOD11A2 products, version 6) were used, provided in a sinusoidal grid format as clear-sky mean LST during an 8-day time frame. The MODIS LST product, estimated using a generalized split-window algorithm under clear-sky conditions, has been widely validated with in-situ measurements, providing a bias less than 0.5 K in most cases [35]. To eliminate effects from the retrieval algorithm errors and clouds, only pixels with high-quality LST values were selected, based on a quality control (QC) flag value of 0. The LST for the city of Ahmedabad is estimated separately for the winter (December, January, and February) and summer (April, May, and June) period. The period from July to November is not analyzable: Since it is the monsoon season, the very frequent cloudy and rainy conditions hinder the use of satellite data [36]. 

Overall, we considered a total of 736 clear sky MODIS images for the 16-year period of 2003 to 2018. For each year, all the images were divided in LST daytime (by averaging the 10:30 and 13:30 data) and nighttime (by averaging 22:30 and 01:30 data), resulting in a total of 46 LST images each year of which 22 for winter (11 daytime and 11 nighttime) and 24 for summer (12 daytime and 12 nighttime).

For the period of 2003 to 2018, data from different sensors and sources were considered: Land use/land cover (LULC) maps, normalized difference vegetation index (NDVI), white sky albedo (WSA), evapotranspiration (ET), population (Pop), and groundwater table level (WT). A brief description of the different datasets follows. 

LULC maps were obtained from the annual global land cover time series of the CCI-ESA having a spatial resolution of 300 m [37]. As for LST, NDVI and WSA were extracted from MODIS products. NDVI was obtained from the MOD13A1 (16-day composite) product, and WSA (bi-hemispherical reflectance) from the MCD43C3 (16-day composite) product. Both NDVI and WSA have a 500-m spatial resolution. Data with the best (QC = 0) and good (QC = 1) quality flags were used.

ET data (https://earlywarning.usgs.gov/fews/product/66#documentation) were obtained from the operational simplified surface energy balance (SSEB) model [38]. The SSEB model has a unique parameterization for operational applications, i.e., pre-defined, seasonally dynamic, boundary conditions that are unique to each pixel. The original formulation of SSEB is based on the hot/cold pixel principles of the SEBAL [39] and METRIC [40] models.

The population dataset from World Urbanization Prospects (WUP) was used to extract Ahmedabad inhabitant information during 1950 to 2035 (https://population.un.org/wup/Download/). The Population Division of the Department of Economic and Social Affairs of the United Nations provides revised estimates and projections of the urban and rural populations of all countries in the world. These data are widely used by many international organizations, research centers, and media.

The Central Water Commission (CWC) has contributed substantially to the collection of hydrological data all over the country (http://cwc.gov.in/). In this study, groundwater level data was used to identify the trend of the water table in the Ahmedabad city. Data from two different tube wells, located in the highly urbanized area at (23.072° N, 72.604° E) and (23.038° N, 72.529° E), were considered.

In addition to these datasets, a field campaign was carried out between 2 and 11 May 2018 to record the LST with an infrared thermometer over cropland/fallowland and built-up sites. The instrument observes the thermal radiation emitted by the target to determine its surface temperature, setting up suitable emissivity values. In this survey, a Testo 810 infrared thermometer [41] was used, which has a measuring range of −30 to 300 °C, with an accuracy of 0.5 °C and a measuring rate of 0.5 s. A total of 74 cropland/fallowland and 84 built-up LST points were measured. 

A summary of the main features of the data used in this work is reported in Table 1.

### 2.3. Methods

The Mann–Kendall test was used to detect the temporal trend of LST datasets, with the magnitude of the trend determined by Sen’s slope estimator [42,43]. After the SUHII computation, a Gaussian surface-fitting method was adopted as an empirical metric to compute the footprint of the heat islands. Then, the potential drivers of the SUHI were analyzed across the years.

#### 2.3.1. LST Trend Analysis

We used the Mann–Kendall (MK) test to detect the temporal trend of LST datasets [42]. The MK test has been extensively used over different hydroclimatic time series and found to be an efficient tool to detect a trend [44,45,46,47,48,49]. It has numerous advantages, for instance, that the analyzed time series are not required to follow a specific linear or nonlinear trend [48]. We calculated the standardized MK test statistics, which displays if a significant or not significant trend is present in the time series at a specific significance level, *p* [45]. In addition, the magnitude of the trend was determined by the Sen’s slope estimator: A positive value indicates a positive trend and vice versa [43]. 

#### 2.3.2. Surface Urban Heat Island Assessment

SUHII is computed by the LST difference between urban and surrounding rural areas [12]. The latter can be considered as a reference and was computed as the mean LST of the rural pixels within a buffer area of about 5 kilometers surrounding the urban boundary (Figure 1). Usually, the warming effect of the heat island is represented by positive SUHII values, while negative values indicate a potential cooling effect in the urban area. An advantage in using the SUHII to describe the thermal behavior of an area is that the LST difference between urban and rural areas dampens the effects of local weather conditions and instrumental error sources.

Since an SUHII decrease can be expected moving away from the city core, with intensities reduced at some distances which would mark roughly the built-up area limit, an estimation of the SUHI spatial extension or footprint can be retrieved modelling the SUHI pattern by a Gaussian surface [36,50,51,52]. The Gaussian surface fitting the spatially distributed SUHI (x, y) (°C) can be modelled as:

(1) $$\mathrm{SUHI}\left(x,y\right)=a_{0}\times \exp\left[-\frac{{\left(\left(x-x_{0}\right)\cos\phi +\left(y-y_{0}\right)\sin\phi \right)}^{2}}{0.5a_{x}^{2}}-\frac{{\left(\left(y-y_{0}\right)\cos\phi -\left(x-x_{0}\right)\sin\phi \right)}^{2}}{0.5a_{y}^{2}}\right],$$

where (x, y) (km) is the pixel location in the map, a_0_ (°C) is the maximum value, a_x_ and a_y_ (km) are the spatial extents, ϕ (deg) is the orientation, and x_0_ and y_0_ is the central location of the fitting surface.

The horizontal cross-section of the Gaussian surface is an ellipse (footprint) with axes a_x_ and a_y_, defining the overall extent of the SUHI. Following the previous works on Gaussian fitting [50,51,52], two main footprint areas can be considered: A_61% (km^2^), defined by the distance from the center where SUHI decreases to 61% (e^−1/2^) with respect to the maximum a_0_; and A_1K (km^2^), the area where SUHI is greater than the fixed threshold of 1 °C.

The Gaussian fitting works well for regular urban shapes and for spaceborne data with MODIS-like pixel size. Since the fitting is represented by a smoothed surface, the representativeness of the Gaussian footprints is drastically reduced when the LST pattern has a great spatial variability [52]. Therefore, a Pearson’s correlation coefficient (*r*) between the SUHI map from MODIS data and the Gaussian surface was computed to assess the capability of the fitting surface to represent the actual pattern. In fact, in the present study, we found that the Gaussian surface is only reliable during nighttime.

**Figure 2:**
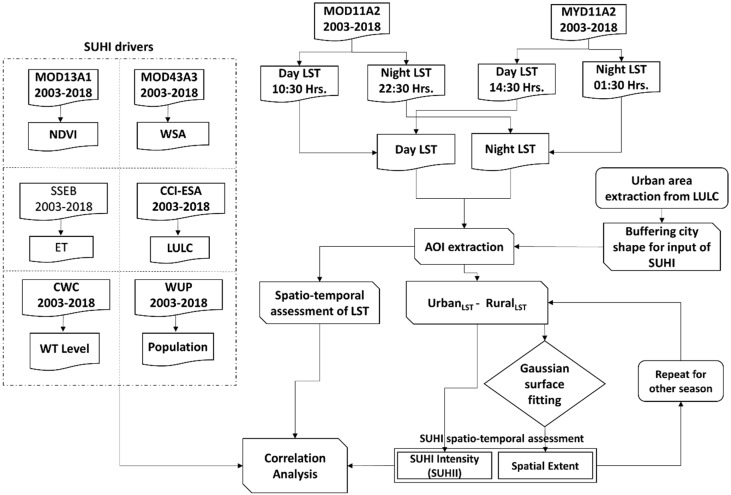
Flowchart for examining the surface urban heat island intensity (SUHII) and their potential drivers. LST, Land Surface Temperature; NDVI, Normalized Difference Vegetation Index; WSA, White Sky Albedo; SSEBop, operational Simplified Surface Energy Balance model; ET, Evapotranspiration; ESA, European Science Agency; LULC, Land Use/Land Cover; CWC, Central Water Commission; GW, Ground Water; WUP, World Urbanization Prospects.

Overall, the adopted methodology is explained with the flow chart diagram in Figure 2.

### 2.4. Potential Drivers of SUHII

It is well known that changes in land use/land cover have significant effects on the SUHI spatial and temporal variations [12]. In our work, cropland and built-up classes were considered as potential drivers of SUHI since these two LULC categories are predominant in our study area. The vegetation index (NDVI), evapotranspiration, and albedo are further important factors that can affect SUHI. The difference between urban and rural areas in NDVI, ET, and WSA (ΔNDVI, ΔET, and ΔWSA) were considered to investigate their driving effect on SUHII. The trend of the total population and groundwater level was also considered in the investigation of the SUHI variations. 

Then, a Pearson’s correlation coefficient, *r*, between the potential driving variables and SUHII across the city and the years was computed.

## 3. Results

### 3.1. Spatial Pattern of LST

**Figure 3:**
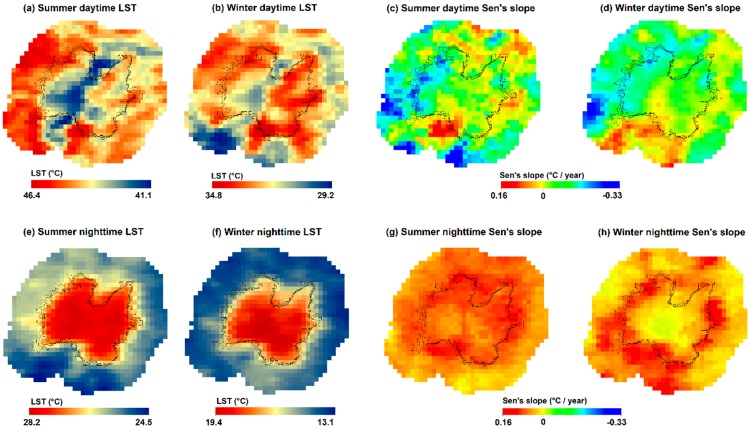
Ahmedabad city and surrounding rural area. Spatial distribution of the LST (average over 2003–2018) for summer daytime (**a**), winter daytime (**b**), summer nighttime (**e**), and winter nighttime (**f**). Sen’s slope of the LST across 2003–2018 for summer daytime (**c**), winter daytime (**d**), summer nighttime (**g**), and winter nighttime (**h**). Dark curves represent the urban boundaries for the years 2003, 2007, 2011, and 2015.

**Figure 4:**
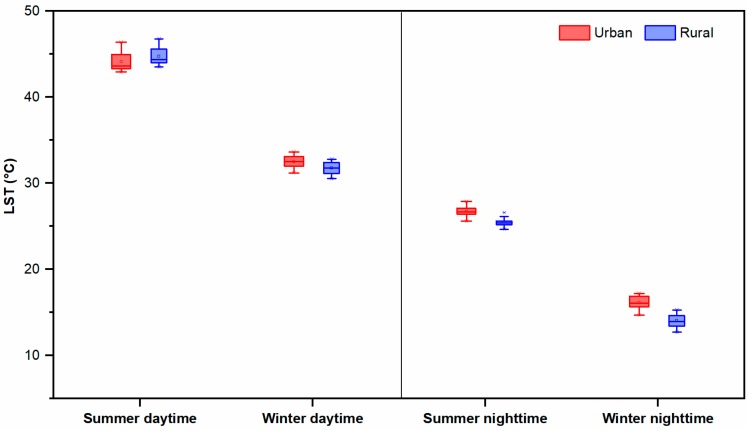
Daytime and nighttime seasonal variation (boxplot) of LST for Ahmedabad city and its surrounding rural area during 2003–2018. The upper and lower whiskers represent the maximum and minimum value of each specific LST dataset; the middle line is the LST average; the box upper and lower edges represent the third and first quartile of the data distribution.

The diurnal, seasonal, and interannual variations of LST from 2003 to 2018 are analyzed in this section. Figure 3 shows the spatial distribution of the average LST over the 16 years for summer/winter and daytime/nighttime, as well as the yearly LST trend (Sen’s slope). The average LST in summer daytime reaches up to 43.72 °C in the urban area and 44.35 °C in the rural area, whereas in winter daytime it is 32.63 °C for the urban area and 32.26 °C for the rural area. The nighttime witnesses comparatively lower LST if compared to daytime: The mean LST in summer is 27.64 °C in the urban area and 25.81 °C in the rural area, while in winter it is 17.85 ° and 14.66 °C. On average, the LST of the urban area exceeds the rural one in the nighttime, prefiguring a positive SUHI effect in both the seasons, while in daytime a variable positive/negative SUHII is expected. In fact, in summer daytime, the surrounding rural area exhibits a higher mean LST with respect to the urban area one. These aspects are summarized in Figure 4, showing the LST variation (boxplot) over the years during daytime and nighttime, in summer and winter.

Also, the daytime LST spatial patterns (Figure 3a,b) exhibit a clear spatial heterogeneity in the urban area with respect to nighttime (Figure 3e,f). This spatial variability within the city area does not allow the daytime Gaussian fitting, as discussed in Section 3.2.

The Mann–Kendall and Sen’s slope estimator tests were performed to detect the trend and slope in the LST data over the 16 years (Figure 3c,d,g,h). For the four maps, 71.5% of the pixels have a significance level of *p* < 0.05, and 22.4% with *p* < 0.1. The slope of the daytime trend ranges between 0.16 to −0.33 °C/year for summer and 0.12 to −0.29 °C/year for winter.

During summer daytime (Figure 3c), the urban–rural transition zone (roughly around the curves) exhibits both decreasing and increasing trends over the years. The surrounding rural area exhibits an increasing LST trend in the northern and eastern part of the city, as well as in a confined southern zone, while the western and southern part has a predominant decreasing trend.

The winter daytime (Figure 3d) has a trend quite similar to summer daytime in the urban and transition zone, even though the latter zone exhibits an LST decay trend in the North not present in Figure 3c. Conversely, the rural zone shows a strong and extensive increasing LST slope in the southern zone. 

The value of Sen’s slope during nighttime ranges between 0.13 to −0.02 °C/year for summer and 0.14 to −0.03 °C/year for winter. During nighttime, a more widespread increasing trend with values greater than 0.1 °C/year is observed for both seasons (Figure 3g,f). During summer nighttime (Figure 3g), the urban area has a positive slope, while it is slightly negative in the city core for winter nighttime (−0.03 °C/year, Figure 3g). The rural areas exhibit different patterns in summer and winter nighttime, with the southern zone again having opposite behaviors. 

Overall, the LST has variable temporal trends in both the urban area and urban–rural transition, also varying seasonally and diurnally, prefiguring that the SUHI intensity will not have a clear increasing trend across the years despite the gradual urban growth. 

### 3.2. SUHI Magnitude and Footprint

**Figure 5:**
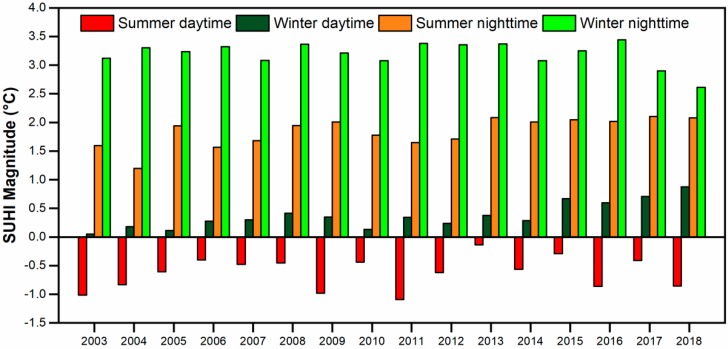
Interannual variation (2003–2018) of the mean SUHI magnitude of Ahmedabad city during daytime and nighttime, in summer and winter.

The mean SUHI (average LST difference between urban and surrounding rural areas), hereafter referred to also as the SUHI magnitude, is shown in Figure 5 as diurnal and seasonal distribution across the years. The summer daytime always has a negative mean SUHII over the city. The mean magnitude over the 16 years during summer daytime is −0.63 °C. The winter daytime has a positive SUHI with a mean magnitude of 0.37 °C, not a significantly high value. The SUHII i more prominent in the nighttime as compared with daytime during both the seasons. The mean SUHI magnitude during summer is 1.84 °C and 3.19 °C in winter. 

As evident from the LST spatial patterns of Figure 3a,b,e,f, the Gaussian fitting, represented by a smoothed surface, cannot be representative of the daytime SUHI, which has a pattern with great spatial variability. In fact, the correlation coefficient, *r*, between daytime MODIS maps and Gaussian surfaces is very low. Conversely, the nighttime SUHI is well fitted by a Gaussian surface, with a mean *r* value of 0.88 during summer and 0.90 during winter.

**Figure 6:**
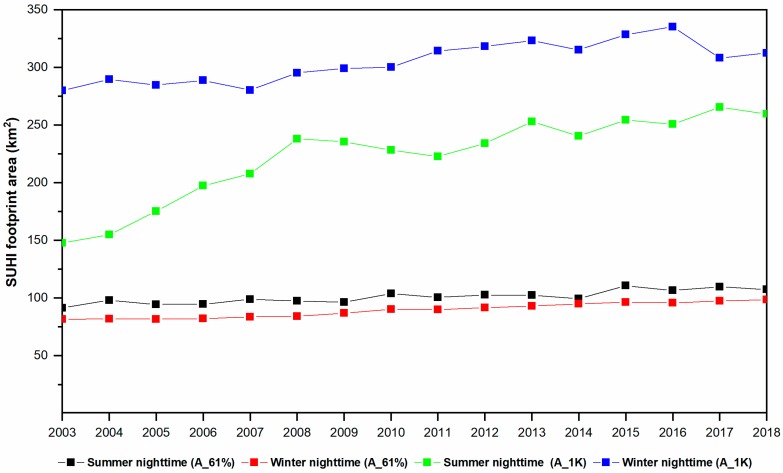
Temporal variation of the nighttime SUHI footprint A_61% and A_1K (km^2^) from 2003 to 2018 during the summer and winter season.

SUHI effects, noticeable in nighttime during both seasons, were then characterized by a footprint estimated by the Gaussian fitting (Section 2.3.2). Figure 6 reports the variation of the nighttime SUHI footprint during 2003–2018. The mean A_61%, i.e., the footprint representing the SUHI modelled area with higher values and therefore interesting the inner city, is 100.91 km^2^ in summer nighttime and 89.37 km^2^ in winter nighttime. The mean A_1K, i.e., the footprint area with modelled SUHII over 1°C, is 222.86 and 304.62 km^2^ during summer and winter nighttime, respectively. 

The A_61% is slightly larger in summer, indicating a wider footprint in the inner city during the warm months. The A_1K area is larger in winter than summer, revealing a spreader distribution of the SUHII during the cold months. A_1K exhibits a clear increasing trend across the years, suggesting that it grasps the urban growth effects well. 

**Figure 7:**
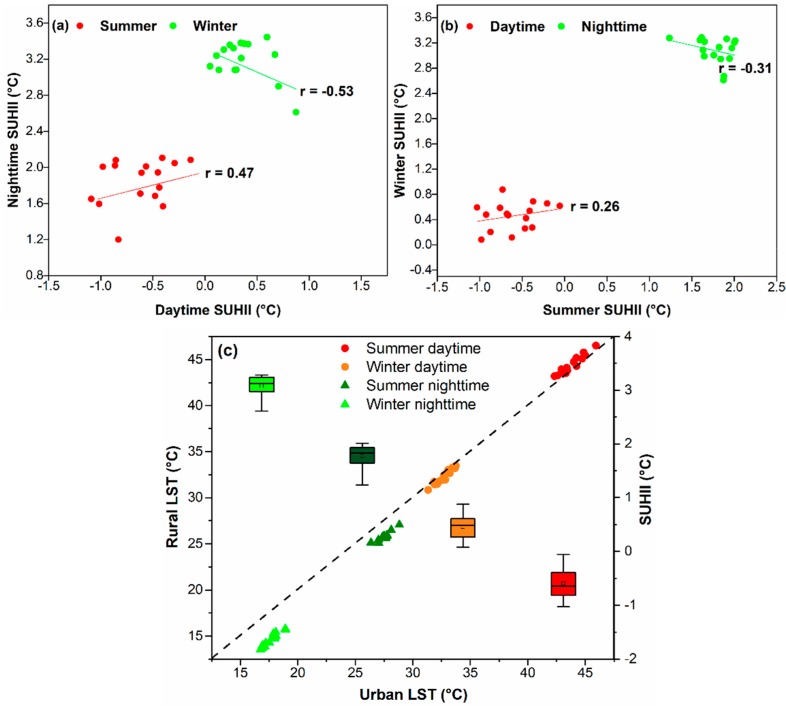
Correlations between day and night mean SUHII for the 16 years (**a**); between summer and winter SUHII (**b**); between urban LST, rural LST, and SUHIIs (**c**). In panel (**c**) the SUHII is represented by a boxplot.

Overall, the relations between the diurnal/seasonal mean SUHII, as well as with urban and rural LST, are shown in Figure 7. For the 16 years, the spatial mean of the daytime SUHII relates positively and significantly with nighttime SUHII in summer (*r* = 0.47) and negatively in winter (*r* = −0.53) (Figure 7a). Meanwhile, a weaker correlation is found between summer and winter SUHII in the daytime (*r* = 0.26) and nighttime (*r* = −0.31) (Figure 7b). A high correlation is observed between the urban and rural LST (Figure 7c). The summer daytime shows the highest LST and winter nighttime the lowest LST, while the SUHII distribution (boxplot in Figure 7c) has a completely opposite behavior, having the highest winter nighttime SUHII, while summer daytime SUHII is negative. 

### 3.3. Rural Area Analysis and Trend of Potential Drivers of SUHI

During summer daytime, the rural area has a higher LST than the urban area, reducing the expected impacts of urbanization on the SUHII, resulting in a negative trend. This unexpected variation of the SUHII can be attributed mainly to the low vegetation cover, the reduced soil wetness, and the dominant bare cropland presence in the surrounding rural area during the pre-monsoon summer season, leading to reduced evapotranspiration [22].

**Figure 8:**
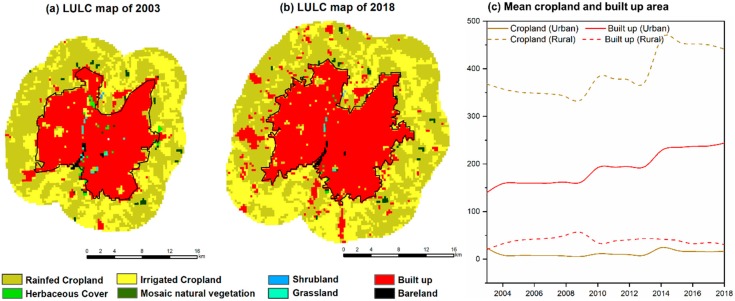
Spatial distribution of LULC classes showing the dominant cropland in the rural area for the year 2003 (**a**) and 2018 (**b**). Panel (**c**) shows the temporal variation of cropland (sum of rainfed and irrigated) and built-up area across the years.

In fact, the majority of rural regions are used as cropland as shown in Figure 8a,b. It consists of 90.74% of cropland and almost negligible mosaic natural vegetation (0.80%). The total cropland area (sum of rainfed and irrigated) in the rural area is around 389.09 km^2^, much higher than the other LULC classes, as reported in Table 2. During the summer dry period, these croplands turn into bare land surfaces, resulting in a higher LST than the built-up area during daytime. In nighttime, the rural LST returns below the urban surface temperature values (Figure 4). 

**Figure 9:**
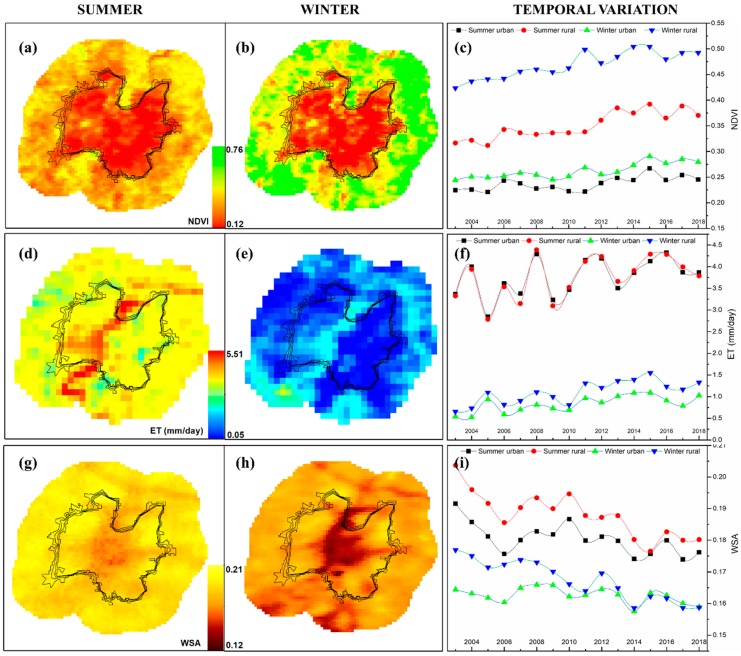
Spatial and temporal variation of NDVI (**a**–**c**), ET (**d**–**f**), and WSA (**g**–**i**) during summer and winter from 2003 to 2018 in both urban and surrounding rural areas. The summer and winter maps show values averaged over the 16 years.

For a better insight of the urban and rural biophysical behavior, Figure 9 shows the spatial and temporal variation of NDVI, ET, and WSA during summer and winter and across the years. Even though NDVI is higher outside the urban boundaries, during summer its values in the rural zone are clearly lower than during winter (Figure 9a–c). Overall, a slight NDVI increasing trend across the years is noticeable.

During summer, the urban zone has a higher ET, especially around Sabarmati River area (Figure 9d); as the mean annual value, summer urban, and rural ET are quite similar, while during winter rural ET is greater than urban ET (Figure 9f). 

In each season, WSA is generally slightly lower in the urban area than in the surrounding rural area, and the mean albedo exhibits a decreasing trend across the years, especially in the rural area (Figure 9g–i). In the urban area, the WSA variations can be ascribed to factors related to the modification over the years of the impervious surfaces (e.g., material, color, ageing) [8]. The WSA temporal decrease in the rural area can be linked to the corresponding NDVI increase. In fact, vegetation covers have low albedo, since the energy stored by plants and foliage is mainly used for their life processes and transpiration [8].

**Figure 10:**
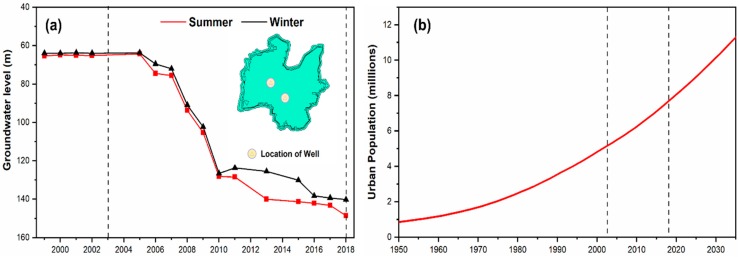
(**a**) Groundwater table level (meters below the surface level) in the Ahmedabad urban area during summer and winter from 1996 to 2018, (**b**) population growth of Ahmedabad city across the years and projections until 2035. Vertical dashed lines represent the study period of 2003–2018

Finally, the temporal trend of WT and Pop is shown in Figure 10. Figure 10a reports the groundwater level as the annual mean of the two well data within the urban area (meters below the surface level), during summer and winter. A clear sinking of WT from about 65 m in 2003 to below 140 m in 2018 is found, which indicates a growing consumption of groundwater in the urban area across the years, more in summer than in winter. 

The population growth of Ahmedabad city across the years, which can be considered an indicator of a built-up class increase, which in turn could impact the SUHI, is reported in Figure 10b. The population increased drastically from 0.85 million in 1950 to 7.68 million in 2018 and it is projected to increase up to 11.29 million in 2035.

### 3.4. SUHI and LST Temporal Correlations with Potential Drivers

**Figure 11:**
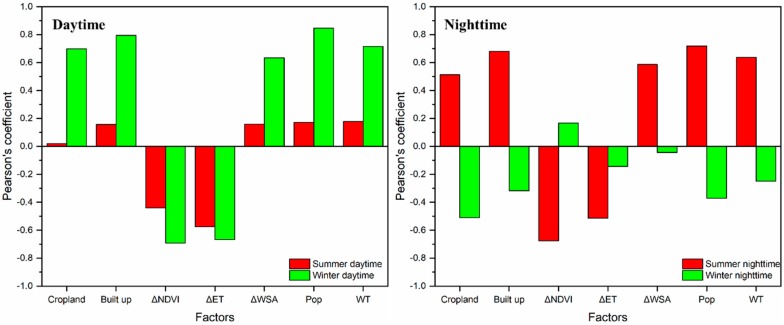
Pearson’s correlation between SUHII (2003–2018) and different potential drivers during daytime and nighttime, in the summer and winter season.

To explore the causes of SUHI variability, seven different parameters were analyzed and correlated: Cropland area (in rural buffer), built up area (within city boundary), ΔNDVI, ΔET, ΔWSA (i.e., NDVI, ET, WSA urban–rural differences), total population (Pop), and groundwater table level (WT). Figure 11 shows the Pearson’s correlation coefficients with the different parameters, averaged to a yearly level. 

During summer daytime, SUHII is not correlated with these parameters, except for ΔET (*r* = −0.58) and, weakly, ΔNDVI (−0.44). Conversely, summer nighttime SUHII is again negatively correlated with ΔNDVI and ΔET, but a significant positive correlation is also found with cropland (0.51), built-up (0.68), ΔWSA (0.59), Pop (0.72), and WT (0.64).

During winter daytime, SUHII exhibits the same significant correlation of summer nighttime, with absolute values greater than 0.63. Conversely, winter nighttime SUHII has very low correlations, except for cropland (−0.51). Despite the prominent magnitude values, winter nighttime SUHII shows weak correlations and the opposite trend with respect to summer nighttime.

As highlighted previously, a negative SUHII is observed during summer daytime, which is mainly due to the large presence of cropland in the surrounding rural area. It is therefore interesting to investigate this behavior also considering the corresponding urban LST, whose values are not biased by the rural area as in SUHII. 

**Figure 12:**
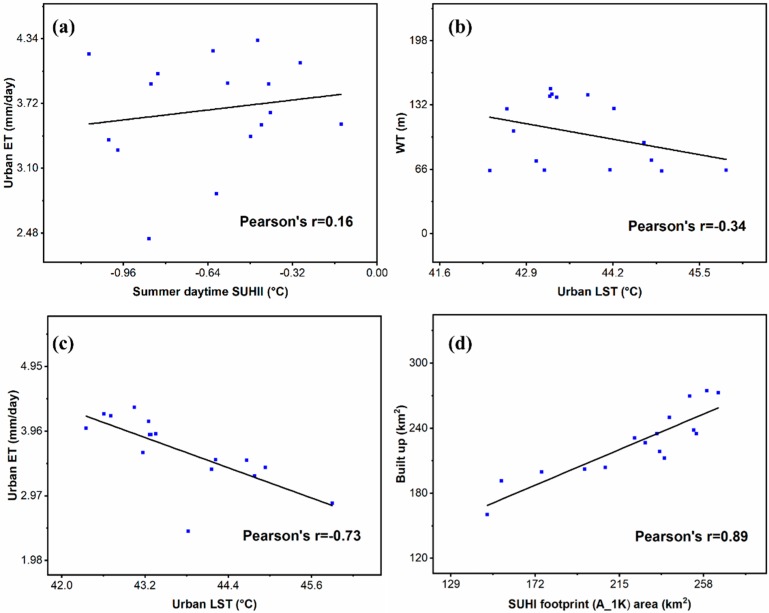
Summer season: scatterplot between (**a**) urban ET and daytime SUHII, (**b**) WT and daytime urban LST, (**c**) urban ET and daytime urban LST, and (**d**) built-up area and SUHI footprint area A_1K.

The summer urban ET is not correlated with the summer daytime SUHII (Figure 12a), while it exhibits a significant negative correlation (−0.73) with the corresponding summer daytime LST within the urban area (Figure 12c). Therefore, in the urban area, an increase of LST is related to an evapotranspiration reduction. Groundwater table levels (WT) within the city area are sufficiently correlated (−0.34) with summer daytime LST (Figure 12b), with the same trend of the urban ET. It suggests that with shallow WT, LST spans both lower and higher values. With deeper WT levels, lower/middle LST values are associated. 

Concerning the SUHI footprints instead of the above magnitudes, Figure 12d shows a very high correlation (0.89) between the summer A_1K footprint and the built-up area across the years. The correlation with the corresponding A_61% is also high (0.83), confirming the ability of the fitted heat island footprints to follow the urban growth. 

### 3.5. In Situ LST from a Field Campaign 

**Figure 13:**
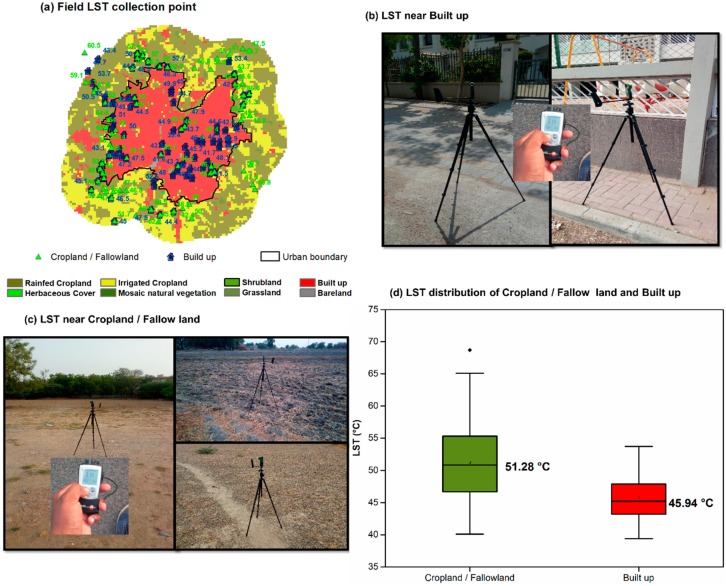
(**a**) Field campaign (2 to 11 May 2018) for LST in situ measurements over built-up (blue symbol) and cropland (green symbol) locations; (**b**) LST recording in built up locations; (**c**) LST recording in cropland/fallowland locations; (**d**) LST distribution of built-up and cropland/fallowland through a boxplot.

A field campaign was carried out to assess and verify the negative SUHII during summer daytime found by using satellite LST data. A total of 74 cropland and 84 built-up points were selected, distributed throughout the study area in different urban and surrounding rural locations (Figure 13a), and the LST was measured. The field survey was performed between 2 and 11 May 2018 in the early afternoon, from 14:00 to 16:00, when maximum solar radiation over the area is present. Figure 13b,c show the pictures of some built-up and cropland/fallowland locations, with the instruments (a tripod with sensors to measure near-surface atmospheric parameters and the infrared thermometer to measure the LST). In the infrared thermometer data processing, we used the emissivity values of 0.98 for cropland and 0.97 for built-up, as reported in the thermometer manual. From Figure 13d, it is evident that the recorded LST over cropland/fallowland was higher than the LST over built-up areas (mean values of 51.28 and 45.94 °C, respectively). This field survey confirms that the surrounding rural area, having more than 90.74% cropland, is warmer than the urban built-up area, thus resulting in an overall negative SUHII over the city during summer daytime. 

The mean LST rural–urban difference is 5.3 °C, comparable with the mean difference found by MODIS data (4.6 °C) for the same days around 13:30, considering the MODIS pixels covering the in situ points.

## 4. Discussion

This research focused on characterization of the surface heating of the urban and surrounding rural region of Ahmedabad city, India. The choice of this topic originated from the major heat wave prevailing over the city during May 2010, with record temperatures that led to 1344 additional deaths registered in May [23]. In the present scenario, it is very important to investigate the diurnal and seasonal variability of the urban heating phenomenon over the city and understand potential driving variables and warming dynamics of urban and rural regions, used to compute the SUHII as an urban–rural LST difference. 

The first objective of the present work was to understand the diurnal, seasonal, and annual behavior of the LST. Overall, the results proved that the LST difference between the urban area and surrounding rural area is clearly greater in nighttime. During summer daytime, the behavior is reversed, thus resulting in a negative SUHI over the city, as for typical arid and semi-arid cities [12,22]. The positive SUHI over Ahmedabad has been reported by various researchers using MODIS and Landsat data [24,25,26], but a discussion on summer daytime negative SUHI is still missing. Consequently, a SUHI characterization over Ahmedabad in a semi-arid climate during summer daytime was provided. This negative SUHI effect during daytime is reported for some urban areas of north-western China [53], the western United States [54], central Asia [55], and South America [20]. All these areas are situated in arid or semi-arid regions, a climate rather similar to the pre-monsoon summer season in Ahmedabad, where the presence in the rural area of large zones of bare croplands with very low vegetation covers and soil moisture reduces cooler transpiration effects [22,55,56]. In fact, in summer daytime, a low soil humidity (about 10%) is present in Ahmedabad (https://mosdac.gov.in/swi/), slightly lower in the rural area than in the urban area.

Considering that cropland usually has higher LST than built-up areas, Kumar et al. [57] found that more than 60% of Indian urban areas experienced a negative summer daytime SUHI. Furthermore, the impact of irrigation on the SUHI variations is significant, and rural areas that are extensively irrigated generally experienced a positive SUHI effect. During nighttime, an intensification of the SUHI occurs across 90% of Indian cities [57]. 

Despite the city growth across the years, the SUHI magnitude in Ahmedabad, as well as the LST, does not have a clear temporal increasing trend, while an evident enlargement of the SUHI spatial footprint was found. These findings suggest that anthropogenic activities and heat emission (e.g., air conditioning systems, energy use in transportation, industrial heat generation) in the city area are important contributor to urban warming [58,59] and have more effects in space that in time. 

The second aim of the work was to explain the potential variables affecting the diurnal and seasonal variations of SUHII. The SUHII is driven by various factors that can be grouped in LULC distribution and changes over the study area, urban site characteristics, and landscape configuration [12]; the more-investigated drivers are related to the vegetation activity, surface albedo, agricultural pattern, and population density [22,60,61]. In the present study, different potential variables were analyzed, i.e., cropland area, built-up area, ΔNDVI, ΔET, ΔWSA, population, and depth of water table. 

During summer and winter daytime, SUHII was negatively correlated with ΔET, meaning that an increase of SUHI intensity is related to a reduction of the urban ET or a rise of the rural ET. These results are confirmed in [22], where the authors point out that the ET increase in the city area, due to higher water use and gardening with irrigation, results in a negative SUHII during summer daytime, while the ET increase in rural regions during winter results in a positive SUHII, in agreement with our outcomes. The same negative correlation for summer and winter daytime is found with respect to ΔNDVI, indicating that urbanization usually leads to a reduction of vegetation activity. A study in Guangzhou, China found that a decrease of 16% of vegetation from 1990 to 2007 resulted in an increase of LST by 2.5 °C [62].

Except for ΔNDVI and ΔET, summer daytime SUHII is not correlated with the other parameters: This fact can be ascribed to the negative and highly variable magnitudes of summer daytime SUHI that are not able to relate to them. Conversely, significant positive correlations of winter daytime and summer nighttime with cropland, built-up, ΔWSA, population, and WT are found. In winter, cropland LST is lower than urban LST, due to irrigation and plantation growing, and rural LST is lower during nighttime. Urban regions with lower albedo, as spatially and temporally found in the Ahmedabad city core, tend to increase the diurnal LST and have more energy for releasing at night [31]. The population growth, acting as an indicator of the built-up sprawl, positively impacts the SUHII only in winter daytime and summer nighttime. In [20], SUHI in South American arid cities exhibited an insignificant correlation with the population both day and night for all seasons. A positive impact of WT on SUHI means that urban warming causes greater water consumption in the city. A research proved that a daily temperature rise of less than 1 °C can cause a monthly water use growth of about 1000 liters for a typical unit family [63]. In Ahmedabad, a study showed that the demand of 400 million liters per day (MLD) of water in 1986 has reached 760 MLD at present. By the year 2031, the projected population growth will lead to the requirement of more than 1600 MLD of water [64]. 

From this standpoint, the results found within the city area considering the biophysical factors of ET and WT and their relationships with summer daytime urban LST, whose values are not biased by the rural area as in SUHII, are very interesting. The urban cooling is strongly correlated to an increase of the urban ET that can be ascribed to the effects of human consumption of water and to the enhanced evapotranspiration from trees and grasses in the city area, thus resulting in a latent and sensible heat flux modification at the surface [22]. 

Overall, city planners should consider the environmental effects of water consumption when they evaluate urban growth strategies, and more general conclusions can be drawn by considering a denser distribution of tube wells. Other limitations and uncertainties remain in the study. For instance, winter nighttime SUHII shows weak correlations and with opposite trends with respect to summer nighttime, suggesting possible more complicated mechanisms underlying SUHII at winter nighttime. For a specific parameter, the variable correlations suggest how a driver can have significant effects or not on SUHI depending on the season and time. Such variations are also different depending on the arid city’s location [20,32]. Also, other important variables may influence SUHI, such as thermal inertia and soil moisture, which were not considered in this study due to a lack of data. Further systematic analyses of the SUHI with these added variables may come up with a better scenario of the SUHI phenomenon.

## 5. Conclusions

This work investigated the variability of LST and SUHI over Ahmedabad city and study of last 16 years (2003–2018) confirmed that the city behaves like a semi-arid urban area, with a mean negative SUHI during summer daytime. This negative intensity is ascribed to the low vegetation activity in the rural area, dominated by croplands turning into bare lands during the pre-monsoon summer months.

Computing the SUHI as the LST difference between the urban area and rural surroundings, variations of SUHII across seasons or years are a consequence of warming/cooling dynamics of both urban and rural areas. A SUHI negative patterns in semi-arid cities is usually ascribed to a warming trend of the rural areas instead of urban heat-alleviation strategies. Therefore, a focused investigation of SUHI and LST, the latter not biased by the rural area temperature as in SUHII, was carried out to better understand the heating dynamics within the Ahmedabad city area. The main novelty of this work was the use of data from different sources and sensors, both space-based and ground-based, not previously explored together in the literature. Also, groundwater consumption data were used for the first time in the SUHI and LST analysis and proved useful for an in-depth investigation of the warming trend in semi-arid cities.

An added value for this work was also the LST field campaign, which provided punctual observations densely distributed over the study area, which is rarely considered in the huge literature dealing with SUHI monitoring from spaceborne sensors. In fact, considering MODIS LST data with 1-km pixel size, in situ measurements can provide detailed information that low spatial resolution data cannot gather, as well as verifications of the LST retrieved from satellite observations.

The last observation concerns the methods for measuring the SUHI: From the literature review they can be grouped into three categories: LST difference between the urban and surrounding reference areas, LST as a proxy of SUHI, and non-parametric/statistical models [12]. In this study, facing the issue of the summer SUHI in semi-arid regions, the application of the urban–rural difference method must be associated with the analysis of LST variations in the city area and their relationships with biophysical factors to help urban planners to formulate site-specific mitigation strategies.

## Figures and Tables

**Table 1:** Data sets used in this study.

No.	Data	Source	Time	Spatial Resolution
1	LST	MODIS (MOD11A2 and MYD11A2)	2003–2018(8-day)	1 km
2	ULC	Climate Change Initiative (CCI) ESA	2003–2008 (Yearly)	300 m
3	NDVI	MODIS (MOD13A1)	2003–2008 (16-day)	500 m
4	ET	SSEB model	2003–2018 (Monthly)	1 km
5	WSA	MODIS (MCD43A3)	2003–2018 (16-day)	500 m
6	WT	Central water commission (CWC)	1996–2018 (Yearly)	Point data
7	Pop	World Urbanization Prospects (WUP)	1950–2035 (Yearly)	City level
8	In situ LST	Infrared thermometer	2–11 May 2018	Point data

**Table 2:** Mean area over 2003–2018 of the different LULC classes in the urban and surrounding rural zones.

LULC	Urban Zone		Rural Zone	
	Area (km^2^)	Area (%)	Area (km^2^)	Area (%)
Cropland	15.47	7.33	389.09	90.74
Herbaceous cover	1.12	0.53	0.71	0.16
Mosaic natural vegetation (tree, shrub, herbaceous cover) (>50%)/cropland (<50%)	0.42	0.20	3.42	0.80
Shrubland	0.02	0.01	0.25	0.06
Grassland	1.24	0.59	0.01	0.00
Built up	191.25	90.63	35.06	8.18
Bare land	1.50	0.71	0.27	0.06
